# Polyacetylene From *Dendropanax morbifera* Alleviates Diet-Induced Obesity and Hepatic Steatosis by Activating AMPK Signaling Pathway

**DOI:** 10.3389/fphar.2018.00537

**Published:** 2018-05-23

**Authors:** Myung-Ji Kang, Eun-Bin Kwon, Hyung Won Ryu, Seoghyun Lee, Jae-Won Lee, Doo-Young Kim, Mi Kyeong Lee, Sei-Ryang Oh, Hyun-Sun Lee, Su Ui Lee, Mun-Ock Kim

**Affiliations:** ^1^Natural Medicine Research Center, Korea Research Institute of Bioscience and Biotechnology (KRIBB), Daejeon, South Korea; ^2^Department of Pharmacology, Chungbuk National University, Cheongju, South Korea; ^3^College of Bioscience and Biotechnology, Chungnam National University, Daejeon, South Korea

**Keywords:** *Dendropanax morbifera*, obesity, hepatic steatosis, triglyceride, AMPK, polyacetylene

## Abstract

The extract tea of *Dendropanax morbifera* is popular beverages in Korea, and their preventive and therapeutic roles in metabolic disorders have been reported. However, the molecular mechanism has not been studied despite the known efficacy of *D. morbifera*. Eleven fractions (fr.1–fr.11) were divided by MPLC to find the active compound. Among them, Fr.5 was superior to others in that the inhibitory efficacy of *de novo* triglyceride (TG) biosynthesis. NMR analysis revealed that Fr.5 is composed 98% or more (9*Z*,16*S*)-16-hydroxy-9,17-octadecadiene-12,14-diynoic acid (HOD). Treatment of HOD diminished oleic acid (OA)-induced TG accumulation in HepG2 hepatocytes and differentiation of 3T3-L1 preadipocytes by activating LKB1/AMPK. In addition, we determined the effect of the oral administration of the extract of *D. morbifera* on obesity and hepatic steatosis in high-fat diet (HFD)-induced obese mice. This study proved that *D. morbifera* containing HOD, the active substance, can show preventive or therapeutic efficacy on obesity and hepatic steatosis through the targeting LKB1/AMPK axis.

## Introduction

Obesity is one of the major health problems accompanied with commodity such as fatty liver, hyperlipidemia, Type 2 diabetes, cardiovascular disease and cancer (Williamson, 2017). Obesity is characterized by an increase in the number and size of adipocytes in adipose tissue. Adipocytes play a key role in energy homeostasis the way that they store excess energy in the form of TG and release in the form of glycerol and fatty acids (Engin, 2017; Wang et al., 2017); therefore, impaired adipocyte functions causes metabolic disorders. In that, reducing the TG levels can be a strategy to treat obesity and related complications (Chen and Farese, 2005). Fibrate, nicotinic acid and omega-3 unsaturated fatty acids are used to lower TG levels in humans (Feingold and Grunfeld, 2000). However, it is necessary to develop an effective TG-lowering agent that is harmless and effective for long-term use.

AMP-activated protein kinase is a sensor protein that monitors intracellular energy status and regulates the uptake and metabolism of fatty acids. AMPK stimulates energy-producing processes such as cellular energy uptake, fatty acid oxidation, glycolysis and ketogenesis and inhibits energy-consuming processes such as lipogenesis, protein synthesis and gluconeogenesis (Ha et al., 2011; Mihaylova and Shaw, 2011; Hardie, 2013; Herzig and Shaw, 2017; Kjobsted et al., 2017; Lin and Hardie, 2017). Therefore, AMPK has been highlighted as a target for the control of metabolic disorders such as obesity and type 2 diabetes.

*Dendropanax morbifera*, an endemic species in Korea, belong to family Araliaceae and can be found in the south-western part of South Korea. Extracts from different parts of *D. morbifera* have had a long history of use in traditional medicine for the treatment of headache, infectious disease and skin disease in Korea (Park et al., 2004; Lim et al., 2015). *D. morbifera* also traditionally used as a varnish, but today they more used as extract tea for the purpose of multi-disease preventive care. Because the main effect of its expectations today is anti-obesity and anti-diabetes, we would like to prove the believes scientifically and mechanistically. In this study, we identified HOD as a result of activity-quieded assay showing the inhibitory activity of TG biosynthesis in DM. HOD reduced intracellular TG levels in HepG2 and 3T3-L1 cells via AMPK–dependent manner. In HFD-induced obese mice, oral administration of DM significantly ameliorated obesity and hepatic steatosis showing meaningful improvement of various obesity-related indicators. We hence suggest that *D. morbifera* and HOD can be useful materials for development of health functional foods and health supplements for obesity and hepatic steatosis management.

## Materials and Methods

### Reagents and Materials

Insulin, 3-isobutylmethylxanthine (IBMX), dexamethasone, orlistat, Nile red, BODIPY 493/503, Hoechst 33342, and Oil Red O were purchased from Sigma-Aldrich (St. Louis, MO, United States). Anti-AMPKα, anti-ACC, anti-phospho-AMPKα (Thr172), anti-phospho-ACC (Ser79), anti-phospho-LKB1 (Ser428), and anti-LKB1 antibodies were obtained from Cell Signaling Technology (Beverly, MA, United States). Anti-tubulin antibodies were purchased from Calbiochem (Millipore, United States).

### Plant Material

**FIGURE 1:**
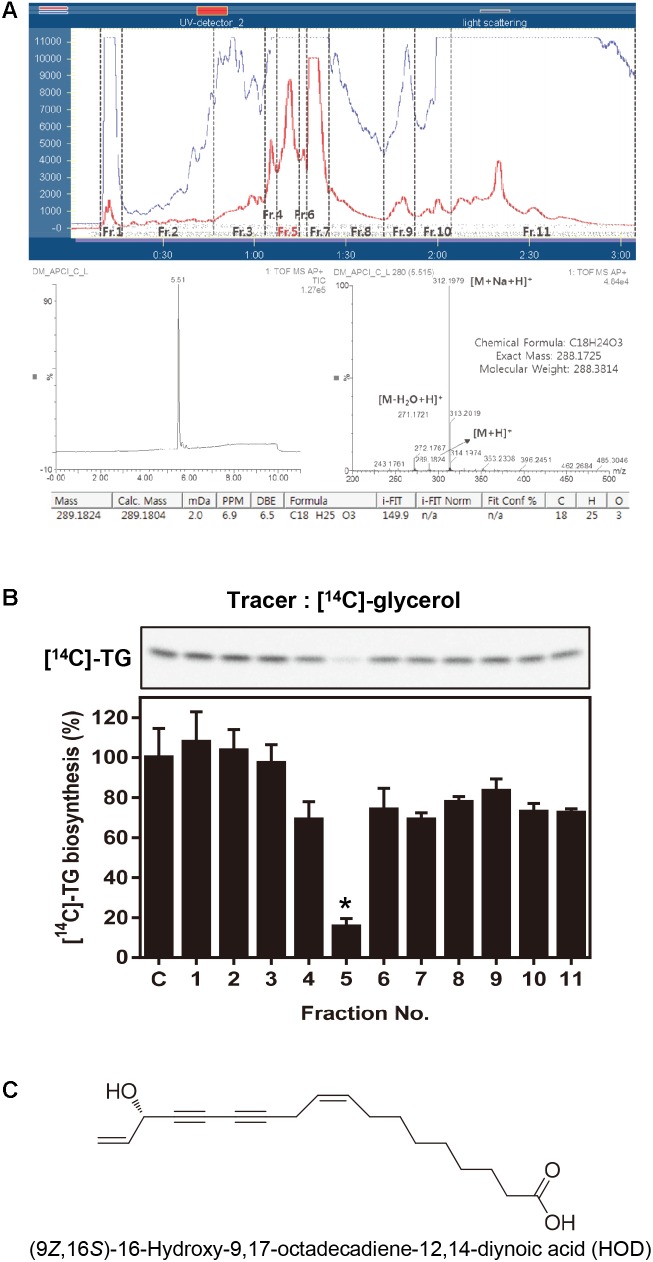
Activity-guided fractionation and identification of HOD. **(A)** Sepbox separation and UPLC-CAD chromatogram data. **(B)** 30 μg/ml of eleven fractions were treated in HepG2 cells and evaluated newly biosynthesized TG. Lipid profile analyzed by TLC using [^14^C]-glycerol as radiolabeled substrates. **(C)** Chemical structure of HOD. Significance: ^∗^*p* < 0.05 vs. control.

*Dendropanax morbifera* leaves sample were cultivated at Bogildo farm in Wando-gun, South Korea (N 34°16′ 20.31^′′^, E 126°54′ 78.26^′′^), in June of 2014 and identified by Dr. Joon-Ku Lee. A voucher specimen (KRIB 0052753) of this raw material is deposited in Korea Research Institute of Bioscience and Biotechnology (KRIBB). HOD was isolated from dried leaves of *D. morbifera* as described previously (Lee et al., 2017). Briefly, the extracts (5.3 g) were separated by Sepbox^®^ 2D-5000 (Sepiatec, Berlin, Germany) using reversed-phase silica gel (Grace Davisil^®^ C18, 50 × 500 mm, 10 μm, Columbia, MD, United States). The lading sample was transferred to the injection column which was flushed with water to remove any water soluble primary metabolite (sugar and amino acid, etc.). The flow rate was 30.0 mL/min using H_2_O as a mobile phase A and MeOH as a mobile phase B. The gradient method was: 0.0 min, 10% B; 0.0–180.0 min, 10–100% B; 180.0–200.0 min, 100% B. The process of fractionation (Frs. 1–11) was monitored and detected by UV (254 nm) and ELSD detectors (**Figure 1A**). This MPLC procedure was repeated 14 times using the same conditions before further isolation. Fraction 5 (2.7 g) enriched with HOD was further separated with a YMC-Pack-ODS AQ column using a gradient of MeOH-H_2_O (0.0 min 50%: 2.0 min, 50%; 10.0 min, 75%; 30.0 min, 90%; 32.0 min, 100%; 35.0 min,100%) to give HOD (710.6 mg). UPLC chromatograms, MS and NMR spectral data of isolated compound are shown in the Supplementary Figures 1–3 and published previously (Lee et al., 2017). Please refer to Supplementary Figure 4 for evaluation of HOD stability with light, storage temperature and storage period.

### Cell Culture and Adipocyte Differentiation

HepG2 cells and 3T3-L1 preadipocytes were obtained from the American Type Culture Collection (ATCC). HepG2 cells were cultured in high glucose-Dulbecco’s Modified Eagle Medium (DMEM, Welgene, Korea), supplemented with 10% fetal bovine serum (Gibco, United States), 1% penicillin-streptomycin (Gibco, United States) at 37°C, 100% humidity and 5% CO_2_. 3T3-L1 cells were maintained in high glucose DMEM containing 10% calf serum in humidified atmosphere of 5% CO_2_ at 37°C. The differentiation of the preadipocytes was induced 2 days post-confluence (day 0) by adding 10% FBS, 0.5 mM IBMX, 1 M dexamethasone and 1 μg/ml insulin (MDI) for 2 days. On day 2, the medium was replaced with DMEM containing 1 μg/ml insulin only. The media was changed every 2 days. On day 7, fully differentiated cells were harvested for stained with Oil Red O dye reagent.

### Determination of *de Novo* TG Biosynthesis

HepG2 cells were incubated with various concentrations of HOD in the presence of [^14^C] glycerol (0.6 μCi) or [^14^C] acetate (1.25 μCi) in serum-free medium for 6 and 2 h, respectively. Cell were washed twice with PBS, and Intracellular lipids were extracted with hexane: isopropanol (3:2, v/v). Total lipids were separated on a thin-layer chromatography by using hexane: diethyl ether: acetic acid (80: 20: 1, v/v/v) and visualized by exposure to a bio-imaging analyzer (FLA-7000, Fuji film).

### Staining and Microscopy

HepG2 cells were seeded in μ-slide plates (μ-slide 8 well, Ibidi, United States). The cells were incubated with or without OA for 16 h. Then, cells were fixed with 4% paraformaldehyde and stained with Nile red (20 ng/ml), BODIPY 493/503 (10 μg/ml) or Hoechst 33342 (10 μg/ml) dye for 15 min. The stained cells were washed PBS and analyzed under a fluorescent microscope (Nikon, Japan).

### Oil Red O Staining

HepG2 cell were pretreated with various concentrations of HOD for 1 h followed by 150 μM OA for 24 h. After incubation, cell were reacted with Oil Red O working solution (0.5% in 60% isopropanol) for 20 min. After rinsing with water, the stained cells were photographed under a microscope. Intracellular Oil Red O was extracted with 100% isopropanol. The absorbance at 500 nm was measured using microplate reader (Epoch, Biotek, United States).

### Western Blot Analysis

Cell were washed with cold PBS and lysed with lysis buffer (Pro-Prep, iNtRON) in ice for 30 min. After centrifugation (13,200 rpm, for 25 min at 4°C), collected supernatants were quantified using the Bredford method. Equal amount of proteins of each sample was electrophoresed on 8% to 12% SDS–PAGE gels and transferred onto polyvinylidene difluoride membranes (Millipore). After incubations with appropriate primary and secondary antibodies, immune complexes were detected by chemiluminescence regent (Thermo).

### *In Vitro* AMPK Kinase Activity

AMPK activity was analyzed using a commercial AMPK kinase assay kit (CycLex, Japan) according to the manufacturer’s instruction. The activity was measured by monitoring the phosphorylated status on IRS-1 at Ser789 by recombinant AMPK enzymes. Conversion of the chromogenic substrate tetramethylbenzidine was quantified by measuring absorbance at 450 nm. AMPK activity was calculated as the difference between the absorbance measured in the absence or in the presence of compound C (10 μM). Recombinant AMPK protein concentrations was used 2 ng/μl.

### Animal Model and Diet

The experimental protocols were approved by the Institutional Animal Care and Use Committee of the Korea Research Institute of Bioscience and Biotechnology (KRIBB-AEC-14172). 40 male C57BL/6 mice (6 weeks old) were housed in a controlled atmosphere (25 ± 1°C at 50% relative humidity) with a 12-h light/dark cycle. The mice were housed with 8 mice per cage and given water *ad libitum*. After acclimation for 1 week, mice were randomly assigned to 1 of 5 groups with equal mean body weight between groups, and fed specific diets as follows: standard chow diet (control, one group, 20% protein, 70% carbohydrate, 10% fat, by Research Diets Inc. #D12450B) or HFD (four groups, 20% protein, 20% carbohydrate, 60% fat, by Research Diets Inc. #D12492). After 4 weeks, when the HFD-fed mice were significantly obese in comparison with the normal diet-fed mice, the treatment regimen was started. Either HOD or orlistat (positive control), dissolved in 0.5% carboxy methyl cellullose (CMC), was administered 5 times per week by oral gavage for 8 weeks. Food intake and body weight were measured 3 times per week. At the end of the experiment, all animals were fasted overnight and sacrificed by CO_2_ asphyxiation. Blood samples were collected from the posterior vena cava and plasma was prepared for biochemical analysis and leptin ELISA. Subcutaneous fat, epididymal fat, and mesenteric fat, and the liver were removed surgically, weighed, and immediately frozen in liquid nitrogen.

### Statistical Analysis

Data are presented as mean ± standard deviation (SD). Statistical analysis was performed using Student’s *t*-test for the *in vitro* experiments. Differences were considered significant at *p* < 0.05 (**^∗^**), *p* < 0.01 (^∗∗^), and *p* < 0.001 (^∗∗∗^). Two-way ANOVA followed by Bonferroni’s multiple comparison test was used for the analysis of body weight and food intake. One-way ANOVA followed by Dunnett’s multiple comparison test was used for fat mass, liver weight, leptin levels, GOT, and GPT analyses. A value of *p* < 0.05 was considered statistically significant.

## Results

### Isolation and Characterization of HOD

In this study, we focused on inhibition of the TG biosynthesis pathway as one of the strategic methods of obesity therapy. HepG2 cells were treated with substrate [^14^C]-glycerol to determine the extent of conversion to [^14^C]-TG, thereby assessing the inhibition of TG biosynthesis. Preliminary experiments have confirmed that DM reduces the conversion of [^14^C]-glycerol to [^14^C]-TG by 39% at the concentration of 30 μg/ml (Supplementary Figure 1). Then, we split the total extract into eleven fractions to identify the putative bioactive molecules (**Figure 1A**). Among the eluted total fractions, fraction 5 was bioactive showing significant inhibition of TG biosynthesis as compared with the control (**Figure 1B**). Further NMR, CAD, and QTof-MS analysis revealed that fraction 5 about 98% consisted of HOD (**Figures 1A,C**). Up to date, there are no reports about the effect of HOD on obesity or specifically on TG biosynthesis mechanism.

### HOD Reduces TG Biosynthesis With a Dose-Dependent Manner

**FIGURE 2:**
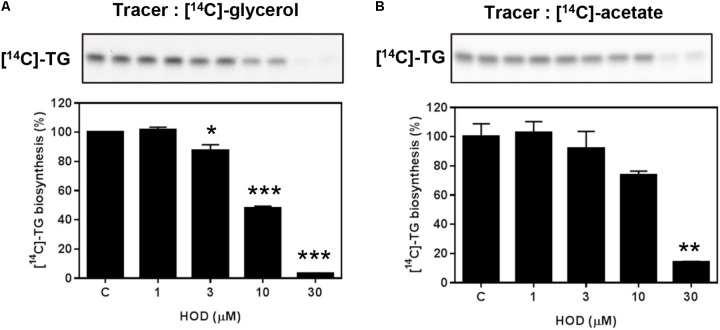
Inhibitory effect of HOD on *de novo* TG biosynthesis. HepG2 cells (5 × 10^5^ cells/ml) were exposed to HOD with various concentrations in the presence of [^14^C]-glycerol for 6 h **(A)** or [^14^C]-acetate for 2 h **(B)**. Newly synthesized intracellular [^14^C]-lipids were extracted and the radioactivity of TG bands were quantified by densitometry using the Multi-Gauge V3 program (Fuji Photo Film Co.). Values are expressed as percentages of control and are means ± SD of the three replicates. Significance: ^∗^*p* < 0.05; ^∗∗^*p* < 0.01; ^∗∗∗^*p* < 0.001 vs. control.

The TG molecule is composed of a glycerol backbone esterified with three fatty acids. The degree of TG biosynthesis in the cells was estimated by using the respective [^14^C]-glycerol and [^14^C]-fatty acid (using [^14^C]-acetate) as a substrate. HepG2 cells were incubated with [^14^C]-glycerol or [^14^C]-acetate for 6 h in the presence of HOD (1, 3, 10 and 30 μg/ml). As shown in **Figures 2A,B**, HOD decreased the incorporation of [^14^C]-glycerol or [^14^C]-acetate into TG in HepG2 cell. The quantitative data presents that treatment of 10 μg/ml HOD showed 52 and 26% suppression of total isotope-leveled TG contents by using [^14^C]-glycerol and [^14^C] -acetate, respectively. Data also presented almost blockage of newly TG biosynthesis as the exposure of 30 μg/ml HOD on HepG2 cells. On the basis of our data suggested that HOD reduces intracellular TG biosynthesis, we further examined to reveal possible modes of action of the active compound.

### HOD Activates AMPK via LKB1

**FIGURE 3:**
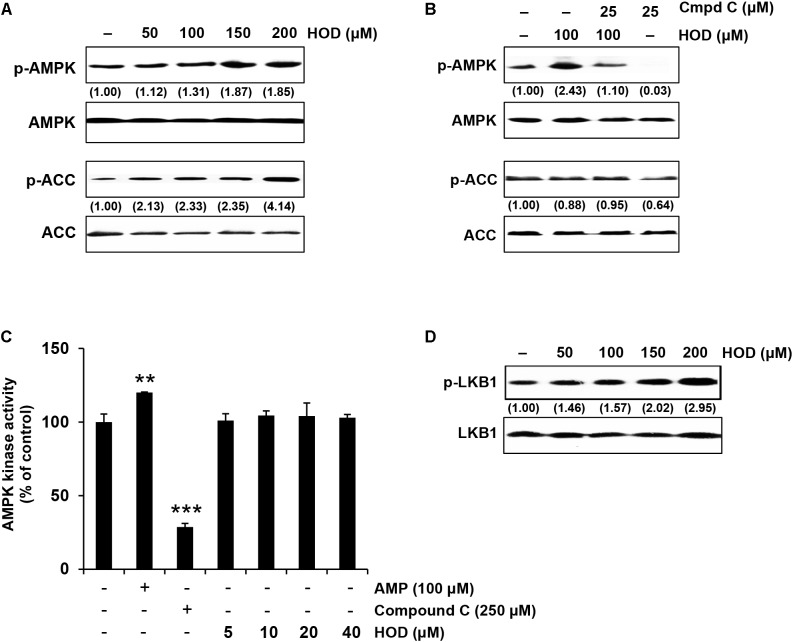
HOD activates the LKB1/AMPK signaling pathway. **(A)** HOD stimulates the phosphorylation at Thr172 of AMPKα and Ser79 of its down-stream target ACC in HepG2 cells. **(B)** Compound C, an AMPK inhibitor, blocks HOD-induced AMPK phosphorylation. The cells were exposed to 100 μM HOD for the indicated period with or without 25 μM compound C. **(C)** AMPK kinase activity using human recombinant AMPK was performed. Significance: ^∗∗^*p* < 0.01; ^∗∗∗^*p* < 0.001 vs. control. **(D)** HOD stimulates the phosphorylation at Ser428 of LKB1.

In order to elucidate the molecular mechanism of HOD on reduction of TG biosynthesis in HepG2 cells, we confirmed the phosphorylation of AMPK, one of the key factors controlling lipid metabolism. As a result, HOD increased AMPK and ACC phosphorylation compared to control dose dependent manner (**Figure 3A**). The effects of HOD on the phosphorylation of AMPK and ACC were reversed by treatment of compound C, a selective AMPK inhibitor (**Figure 3B**). Further, recombinant human AMPK enzyme was used to confirm whether HOD directly activates AMPK enzyme activity. However, as shown in **Figure 3C**, HOD did not directly stimulate AMPK enzyme activity. We had to find the upstream molecule that influenced AMPK activation. LKB1 is one of the well-known activators of AMPK, which phosphorylates and activates AMPK according to changes in AMP/ATP ratio, nutrition, hypoxia, pH, redox state, and creatine/phosphate creatine ratio (Ducommun et al., 2014; Behunin et al., 2015). As expected, treatment of HOD dose-dependently increased the phosphorylation of LKB1 (**Figure 3D**). These data present that HOD activates AMPK through LKB1 but not directly.

### HOD Prevents OA-Induced Cellular Steatosis

**FIGURE 4:**
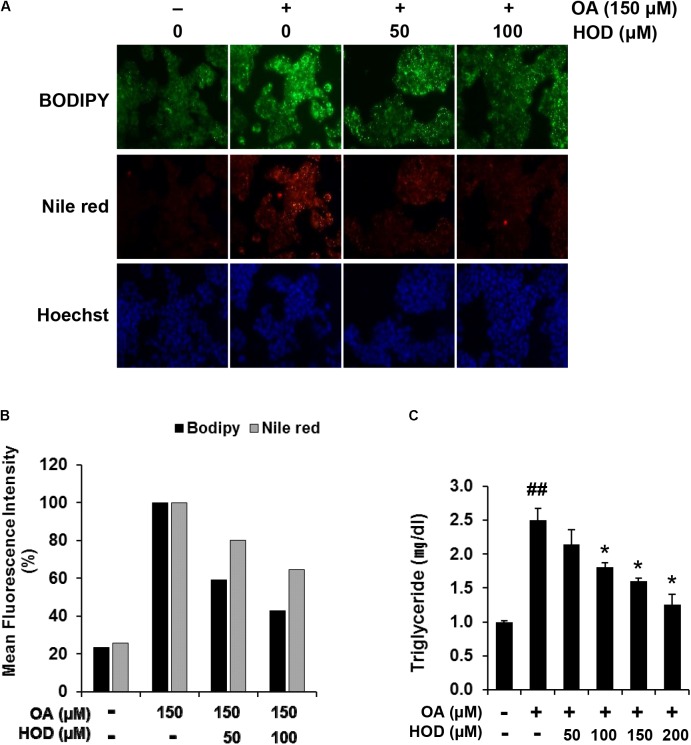
HOD decreases the OA-induced intracellular lipid accumulation in HepG2 cells. **(A)** The cells were pre-treated with indicated concentrations of HOD for 1 h, followed by exposed to 150 μM OA for 24 h. Lipid accumulation was determined by using BODIPY 493/503 and Nile red staining. Nuclei were counterstained with Hoechst 33342. **(B)** Quantitative analysis was performed after BODIPY 493/503 and Nile red staining by using the flow cytometry in the FL-1H and FL-2H channel, respectively. **(C)** Quantification of TG contents by using commercial kit. The bar graphs show the mean ± S.D. of 3 independent experiments (^##^*p* < 0.01 compared with the DMSO control; ^∗^*p* < 0.05 compared with the OA-treated control).

Currently, the studies on *in vitro* cell models of hepatic steatosis use OA to induce cellular steatosis in hepatocytes (Feldstein et al., 2004; Liu et al., 2011). To determine whether HOD attenuated lipid accumulation, HepG2 cells were pretreated with 50 or 100 μM of HOD before the exposure of 150 μM OA for 24 h. The cells were stained with BODIPY 493/503 (green) or Nile red (red) to visualize neutral lipid droplets. As shown in **Figure 4A**, the intracellular lipid contents were reduced significantly by pretreatment of HOD compared to OA alone. Analysis of flow cytometry showed that pretreatment with HOD 50 and 100 μM inhibited lipid accumulation compared to OA treated controls (41 and 57% for BODIPY staining and 19 and 35% for Nile red staining, respectively) (**Figure 4B**). Additionally, the results of quantitative analysis of TG commercial kit showed similar trends to those of the previous results (**Figure 4C**). Collectively, the treatment of HOD decreases OA-induced lipid accumulation in HepG2 cells, suggesting a possibility for preventing the hepatic steatosis.

### HOD Blocks Lipid Accumulation and Increases AMPK Phosphorylation During Adipogenesis

**FIGURE 5:**
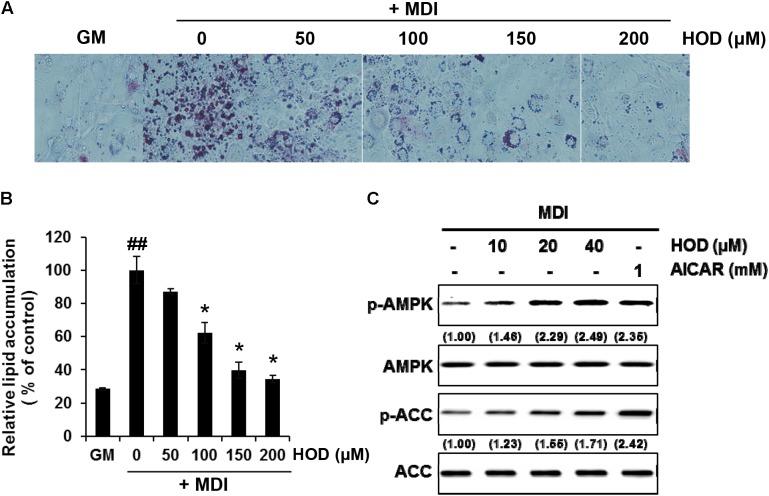
Effect of HOD on early adipogenesis of 3T3-L1 and phosphorylation of AMPK and ACC. 3T3-L1 preadipocytes were differentiated into adipocytes in the presence of specified concentrations of HOD. **(A)** After 7 days, intracellular lipid droplets were stained using Oil-red O, and observed microscopically. **(B)** Quantification of intracellular lipid accumulation. Total lipids stained with Oil red-O were extracted in absolute isopropanol, after which the absorbance of the solution was measured at 500 nm. The bar graphs show the mean ± S.D. of 3 independent experiments (^##^*p* < 0.01 compared with the GM control; ^∗^*p* < 0.05 compared with the MDI-treated alone control). **(C)** Total protein and their phosphorylated form levels of AMPK and ACC were analyzed by western blotting. The numbers at the bottom of the figure indicate the relative band intensity normalized to that of the non-phosphorylated protein (fold-change in comparison with that of the control group).

We investigated the effect of HOD on 3T3-L1 differentiation. Post-confluent 3T3-L1 preadipocytes were treated MDI to initiate differentiation. The medium was changed once in every 2 days with various concentrations of HOD (0–200 μM) for 6 days. At the end of the experiment, morphologic and quantitative analysis of intracellular lipid contents were performed using Oil Red O staining. HOD-treated cells were significantly decreased the number and size of lipid droplets in compared to those of control suggest that HOD could inhibit the adipogenesis. (**Figure 5A**). The inhibitory effect of HOD on lipid accumulation was noted about 50% decrease at 100 μM (**Figure 5B**). Several evidence suggests that AMPK activation can inhibit adipogenesis (Lee et al., 2011; Figarola and Rahbar, 2013); therefore, AMPK is recognized as a target for anti-obesity treatment. Hence, we checked whether HOD amplifies AMPK phosphorylation, which is decreased when 3T3-L1 cells are differentiated. Two-day post-confluent 3T3-L1 preadipocytes were exposed with HOD at the concentrations of 10, 20 and 40 μM for 1 h in the presence of MDI. Forty μM of HOD increased phosphorylation of AMPK and ACC by 2.3- and 2.4-times higher than that of the control group (**Figure 5C**). Collectively, these results suggest that HOD may activate AMPK signaling in 3T3-L1 adipocytes, eventually leading to suppression of adipogenesis.

### DM Improves Obesity and Hepatic Steatosis in HFD-Induced Obese Mice

**FIGURE 6:**
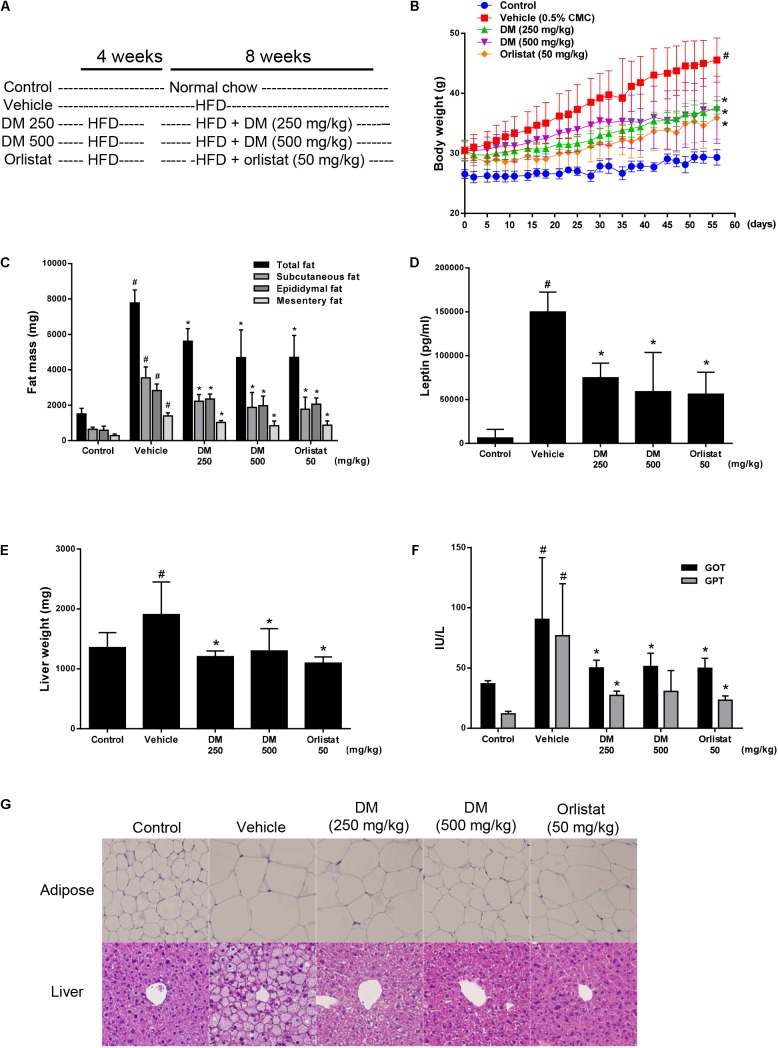
Effects of DM on HFD-induced obesity in C57BL/6 mice. DM (250 or 500 mg/kg) or orlistat (50 mg/kg) were administered by oral gavage for 8 weeks while the mice were fed the HFD. **(A)** Experimental outline. **(B)** Body weight was measured 3 times per week. The group that received HFD alone (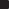) showed steady body weight gain, while the DM-(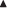 or 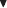) and orlistat-treated (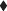) groups showed significantly attenuated body weight gain. **(C)** Adipose tissue weight of subcutaneous, epididymal, and mesentery fat. **(D)** Amount of leptin in plasma measured by ELISA. **(E)** Liver weight. **(F)** Plasma levels of GOT and GPT measured using a chemical analyzer. **(G)** H&E-stained images of liver and subcutaneous adipose tissue samples from the normal diet (control), vehicle-treated, DM-treated, and orlistat-treated groups. The results are expressed as the mean ± SD for each group (*n* = 8). ^#^*p* < 0.05 compared with the control group; ^∗^*p* < 0.05 compared with the HFD-treated vehicle group.

After feeding HFD to C57BL/6 mice for 4 weeks, the body weight of the HFD group was significantly increased in comparison with that of the normal chow diet-fed control group. HFD-fed mice were divided into 4 groups: HFD alone (vehicle), HFD + DM (250 or 500 mg/kg), and HFD + orlistat (50 mg/kg), which were administered for further 8 weeks (**Figure 6A**). Orlistat, an FDA approved anti-obesity drug, served as a positive control. The mice receiving either 250 or 500 mg/kg had a mean weight gain of 7.2 g (*P* < 0.05) or 6.8 g (*P* < 0.05) during the drug intervention period, while the HFD vehicle control group had an average weight gain of 15.1 g. (**Figure 6B** and **Table 1**). The DM-treated groups and the group that received the HFD alone showed similar daily food intake (**Table 1**). Administration of 250 or 500 mg/kg DM significantly reduced the total fat mass, including the subcutaneous, epididymal, and mesentery fat of treated mice in comparison with that of the group that received the HFD alone (**Figure 6C**). In accordance with these results, adipocyte size analysis in subcutaneous adipose tissue by H&E staining revealed that the size of the enlarged adipocytes in the HFD-fed mice was considerably reduced in the DM-treated mice in comparison with that of the normal chow-fed control mice (**Figure 6G**, upper panels). Serum profiles were also analyzed (**Table 1**). DM-administered HFD-mice showed significantly reduced the serum TG level, while the group that received the HFD alone was higher than that of the normal food-fed control group. It is worth mentioning that the TG level of 500 mg/kg DM-administered HFD-mice was even lower than that of the normal food-fed control group, indicating improved lipid homeostasis. Unusually, the total cholesterol level of the group that received DM showed little change compared with the group that received the HFD alone (which was marginally increased in comparison with that of the normal food-fed control group). Leptin production is closely associated with adiposity; therefore, we measured serum leptin levels in mice. The serum leptin level of mice fed the HFD alone was significantly increased by approximately 24-fold in comparison with that of the normal control group. However, 250 and 500 mg/kg of DM significantly attenuated the HFD-induced increase in serum leptin level by approximately 50 and 60%, respectively (**Figure 6D**).

**Table 1:** The changes of body weight, food intake, and serum profiles.

		+ HFD			
	Normal	Vehicle	DM 250 mg/kg	DM 500 mg/kg	Orlistat 50 mg/kg
Initial weight (g)	26.5 ± 0.7	30.5 ± 1.4	30.5 ± 1.7	30.5 ± 1.7	30.4 ± 1.7
Final weight (g)	29.3 ± 1.3	45.6 ± 3.7	37.7 ± 1.1	37.3 ± 5.6	35.9 ± 3.6
Weight gain (g)	2.8 ± 0.7	15.1 ± 2.4^#^	7.2 ± 3.5*	6.8 ± 4.9*	5.5 ± 2.0*
Food intake (g/day)	3.9 ± 0.6	3.7 ± 0.8	3.3 ± 0.8	3.3 ± 0.8	3.8 ± 0.9
Triglyceride (mg/dl)	68.0 ± 43.3	93.0 ± 40.7	54.8 ± 39.7	36.6 ± 13.0*	40.9 ± 17.1*
Total Cholesterol (mg/dl)	119.0 ± 15.9	211.6 ± 31.5^#^	214.0 ± 17.7	194.4 ± 42.5	178.6 ± 15.4
HDL-C (mg/dl)	74.0 ± 10.5	104.3 ± 13.0^#^	115.1 ± 4.7	108.1 ± 19.3	101.5 ± 5.5
LDL-C (mg/dl)	6.0 ± 0.8	9.3 ± 2.4^#^	8.3 ± 2.4	7.8 ± 2.3	6.6 ± 0.9*

*Values are expressed as the mean ± SD (n = 8 per group). Statistical significance compared with the HFD group (One-way ANOVA followed by Dunnett’s multiple comparison test): ^∗^P < 0.05. Statistical significance compared with the control group (One-way ANOVA followed by Dunnett’s multiple comparison test): ^#^P < 0.05.*

A HFD can lead to hepatic steatosis, inflammation, and injury, which can subsequently lead to non-alcoholic fatty liver disease (NAFLD) and steatohepatitis. After 12 weeks of HFD, the liver weight and serum GOP and GTP levels of the mice that received the HFD alone were significantly increased in comparison with those of the control group. These results indicated that hepatic steatosis and injury occurred in the group that received the HFD alone (**Figures 6E,F**). Consistent with this finding, histological examination of the group that received HFD alone via H&E staining showed significant lipid droplet accumulation in the liver (**Figure 6G**, lower panels). Administration of DM at 250 and 500 mg/kg markedly attenuated the HFD-induced increases in liver weight, GOT and GTP levels, and hepatic lipid droplet formation in a dose-dependent manner. These results strongly suggest that DM ameliorates hepatic steatosis and injury in HFD-fed mice. DM showed similar *in vivo* efficacy at 10-times higher concentration than the FDA approved anti-obesity drug Orlistat.

## Discussion

*Dendropanax morbifera* has been used medicinally because of their anti-diabetic and anti-oxidant properties (Moon, 2011; Hyun et al., 2013; Kim et al., 2015). Considerable compounds isolated from *D. morbifera* have been shown to possess physiological activities (Chung et al., 2009; Kim R.W. et al., 2016; Kim W. et al., 2016); however, this study is the first to demonstrate that HOD from *D. morbifera* can contribute to anti-obese efficacy through inhibition of the TG biosynthesis pathway. To tracking the active ingredient, the DM divided by Sepbox into eleven fractions (fr.1–fr.11), and among them Fr.5 is superior to others in that the inhibitory efficacy of *de novo* TG biosynthesis in HepG2 cells (**Figure 1**). Interestingly, Fr.5 is composed 98% or more polyacetylene, and most of polyacetylene was identified as HOD by NMR spectroscopy. A subsequent series of experiments demonstrates that HOD can inhibit intracellular lipid accumulation through the activation of AMPK in HepG2 hepatocytes and 3T3-L1 adipocytes, respectively (**Figures 3**, **5**).

Because AMPK activity is reduced by obesity (Ha et al., 2011), diabetes (Day et al., 2017; Kjobsted et al., 2017), and inflammation (Day et al., 2017), increasing AMPK enzymatic activity has been considered as a therapeutic strategy to improve obesity and hepatic steatosis. Indeed, metformin, an AMPK activator, induces modest weight loss in obese individuals, and improves hepatic steatosis and suppresses liver inflammation in both human and animal studies (Smith et al., 2016). Mechanistically, anti-obesity effect of AMPK is not only include reduction of fatty acid synthesis but also increase of decomposition of TG in adipocytes (Kohjima et al., 2008; Kang et al., 2018). When ACC is phosphorylated by AMPK, which results in attenuating malonyl-CoA synthesis and subsequently leads to increase β-oxidation by activation of enzyme CPT-1, ultimately decreasing TG accumulation (Wang et al., 2014). In addition, AMPK is associated with adipocyte differentiation by modulating adipogenic transcription factors and fatty acid synthesis in 3T3-L1 adipocytes (He et al., 2013). Emerging evidence supported that natural compound sulforaphane (Choi et al., 2014) and ursolic acid (He et al., 2013) as well as AICAR (Giri et al., 2006), an AMPK activator, inhibit adipogenesis by targeting AMPK activation.

AMP-activated protein kinase is a kinase that plays an important role in intracellular energy metabolism and exist in many tissues including liver, brain, adipose tissue, and muscles (Day et al., 2017). Activating AMPK requires increase of AMP/ATP ratios or up-regulation of three known upstream kinases: the LKB1 (Hardie and Alessi, 2013); the calcium-dependent calcium/calmodulin-dependent protein kinase kinase β (CaMKKβ) (Abbott et al., 2009); or transforming growth factor-β activated protein kinase-1 (TAK1) (Zippel et al., 2013). Our study suggests that LKB1 is a major upstream kinase of HOD-induced AMPK activation (**Figure 3C**). Activation of LKB1 essentially requires binding of the scaffold protein MO25 and stabilization by Ste20-related adaptor (STRAD) protein (Behunin et al., 2015). These active trimeric LKB1-STRAD-MO25 complex that has been shown to mediate AMPK Thr-172 phosphorylation in multiple mammalian systems. Therefore, further studies are required on how HOD modulates the LKB1-STRAD-MO25 complex to activate LKB1.

The results of this study are outstanding in that *in vitro* and *in vivo* results can be interpreted consistently. First, we have confirmed in HepG2 hepatocytes that HOD can reduce lipid accumulation by inhibiting intracellular TG biosynthesis (**Figures 2**, **4**). Assuming that HOD is the main compound showing physiological activity in DM, oral administration of DM significantly decreased hepatic lipid droplets and weight, which were increased by HFD (**Figures 6E,G**). Second, the treatment of HOD inhibited differentiation of 3T3-L1 cells and reduced the intracellular lipid accumulation (**Figure 5**). These results are consistent with the following results of a decrease in the total fat mass including the subcutaneous, epididymal, and mesentery fat of DM-administrated mice in comparison with that of received the HFD alone (**Figures 6C,G**). Finally, the activation of AMPK at the cellular level can be interpreted as a result of reduced serum TG concentration, not cholesterol, following inhibition of TG biosynthesis of liver and adipose tissue (**Figures 3**, **5** and **Table 1**).

Through *in vivo* evaluation, this study presents several lines of evidence demonstrating that DM has preventive and therapeutic effect on obesity and hepatic steatosis in HFD-fed obese mice. First, DM reduced body weight gain. Second, the levels of serum leptin, which reflect growth of adipose tissue in obese animals, were significantly lowered by DM treatment, suggesting that DM can improve leptin resistance in HFD-fed mice. Third, DM treatment of HFD-fed mice decreased the adipose tissue mass. Finally, DM reduced hepatic lipid accumulation in HFD-induced obese mice. Moreover, decreased serum levels of GPT and GPT were suggesting that DM attenuated the hepatic injury caused by HFD. The *in vivo* activity of DM showed similar efficacy at 10-times higher concentration than the FDA-approved anti-obesity drug orlistat.

## Conclusion

In conclusion, this study proved that *D. morbifera* can ameliorate the obesity and hepatic steatosis through the targeting LKB1/AMPK pathway. Our findings may provide a basis for future developments of novel therapeutic strategies for obesity and hepatic management, and further studies are needed.

## Author Contributions

M-JK, E-BK, and M-OK designed and performed the experiments. HWR, SL, J-WL, and D-YK performed the experiments. MKL, S-RO, and H-SL analysis and interpretation of the data. HWR isolation, identification, and interpretation of HOD. M-JK, SUL, and M-OK have created and revised the manuscript.

## Conflict of Interest Statement

The authors declare that the research was conducted in the absence of any commercial or financial relationships that could be construed as a potential conflict of interest.

## Abbreviations

AMPK: AMP-activated protein kinase
HFD: high-fat diet
HOD: (9*Z*,16*S*)-16-hydroxy-9,17-octadecadiene-12,14-diynoic acid
LKB1: liver kinase B1
OA: oleic acid
TG: triglyceride
