# Supplementary material for: Polyacetylene From Dendropanax morbifera Alleviates Diet-Induced Obesity and Hepatic Steatosis by Activating AMPK Signaling Pathway
Source: Front Pharmacol. 2018 May 23;9:537. doi: 10.3389/fphar.2018.00537 (PMC5975361; doi:10.3389/fphar.2018.00537)
Supplement: Supplementary file 1 [file Presentation_1.PDF]

# Supplementary information

*UPLC-QTof-MS analysis*

The leaves extract of *D. morbifera* profiling was performed using an ACQUITY UPLC™ system (Waters Corporation, Milford, MA, USA) equipped with a binary solvent delivery manager and a sample manager coupled to a Micromass Q-Tof Premier™ mass spectrometer (Waters Corporation) with an electrospray ionization (ESI) interface with MassLynex V4.1 software. Chromatographic separation was performed using an ACQUITY HSS T3 chromatography column (2.1 × 100 mm, 1.8 μm). The column temperature was maintained at 35°C, and the mobile phases A and B were water with 0.1% formic acid and acetonitrile with 0.1% formic acid, respectively. The gradient elution program was as follows: 0.0-1.0 min, 10% B; 1.0-7.0 min, 10-98% B; 7.0-9.0 min, 98% B; and then held for 2.0 min before returning to the initial conditions. The injection volume was 1.0 μL, and the flow rate was 0.4 mL/min. The mass spectrometer was operated in negative ion mode. Leucine-enkephalin was used as the reference compound (*m/z* 554.2615 in the negative mode). $N_2$ was used as the desolvation gas, and the desolvation temperature was set to 350°C at a flow rate of 500 L/h and a source temperature of 100°C. The capillary and cone voltages were set to 2300 and 35 V, respectively. The Q-Tof premier was operated in "V" mode with 9000 mass resolving power. The data were collected for each test sample from 100 to 1500 Da with a 0.25 s scan time and a 0.01 s interscan delay over a 35 min analysis time.

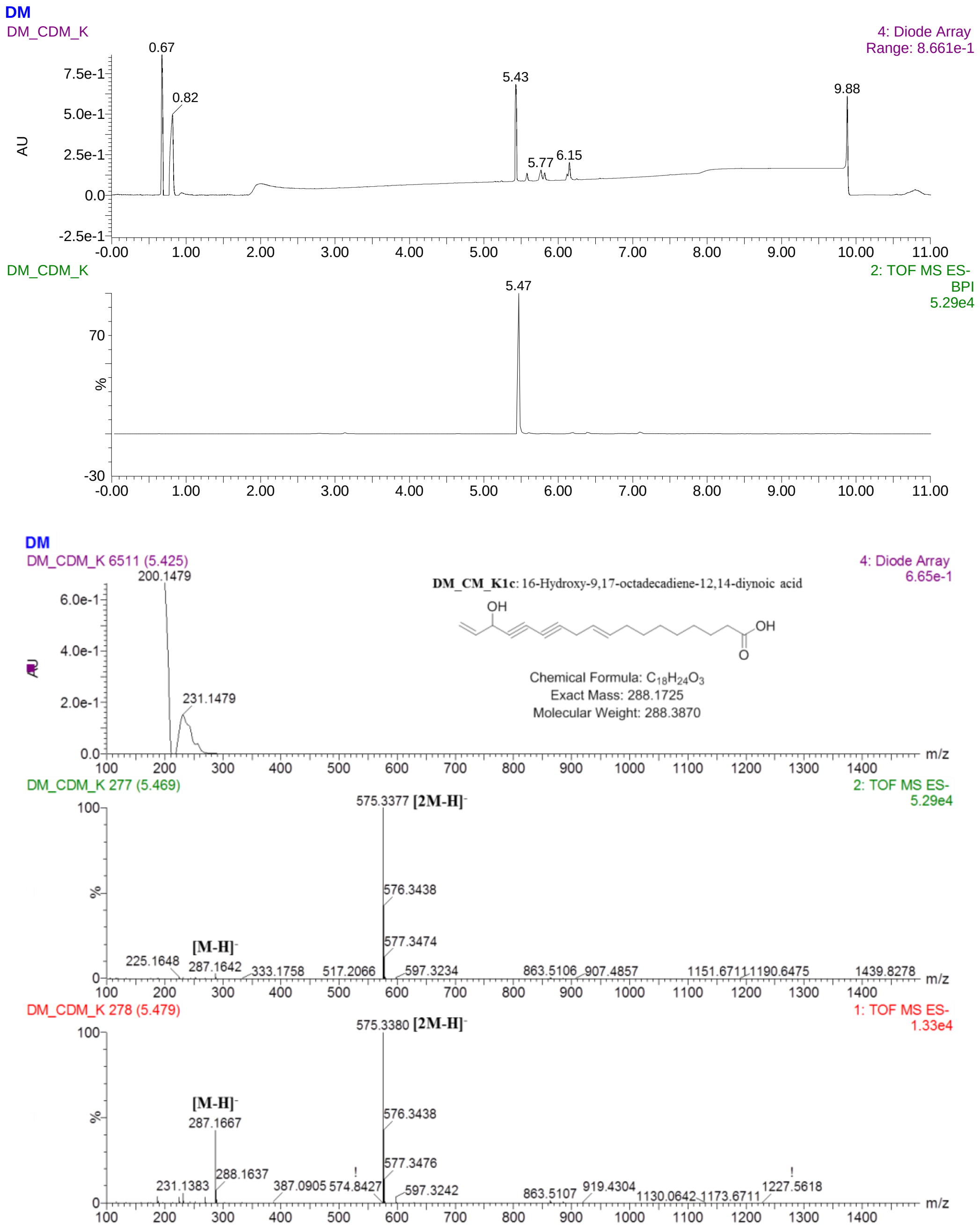


**Supplemental Figure 1.** UPLC-QTOF-MS and HREIMS data of (9*Z*, 16*S*)-16-Hydroxy-9,17-octadecadiene-12,14-diynoic acid.

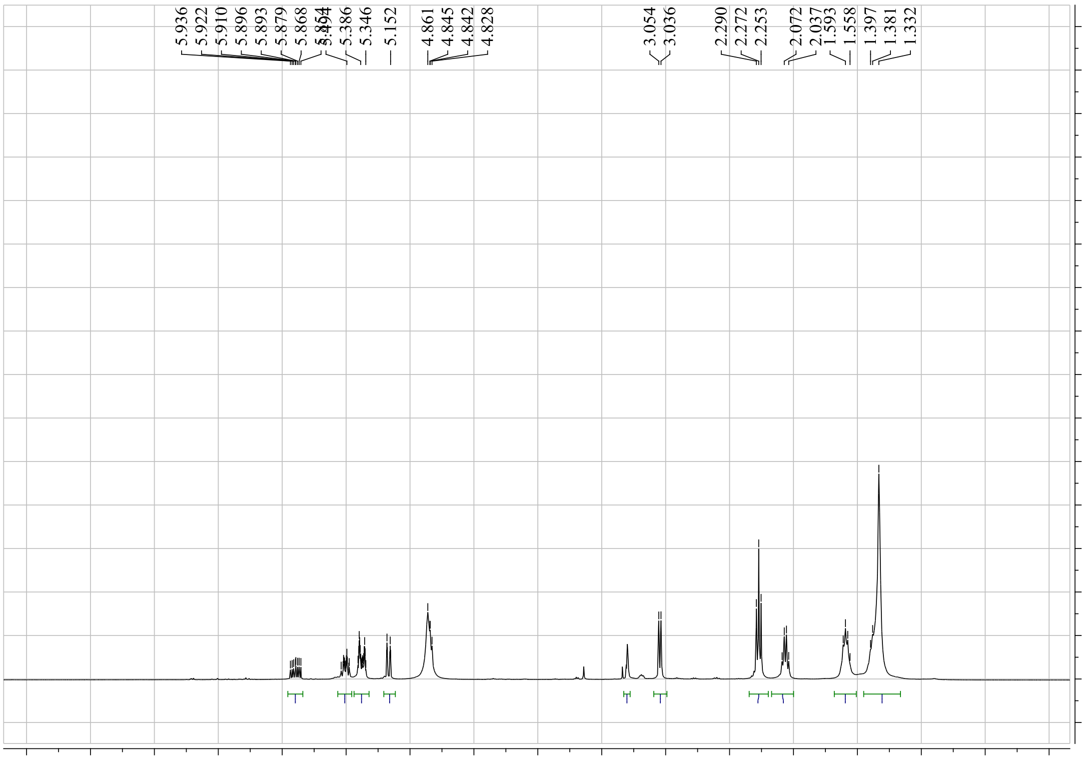


**Supplemental Figure 2.** $^{1}$H-NMR spectrum of (9*Z*, 16*S*)-16-Hydroxy-9,17-octadecadiene-12,14-diynoic acid (400 MHz, $CD_3OD$).

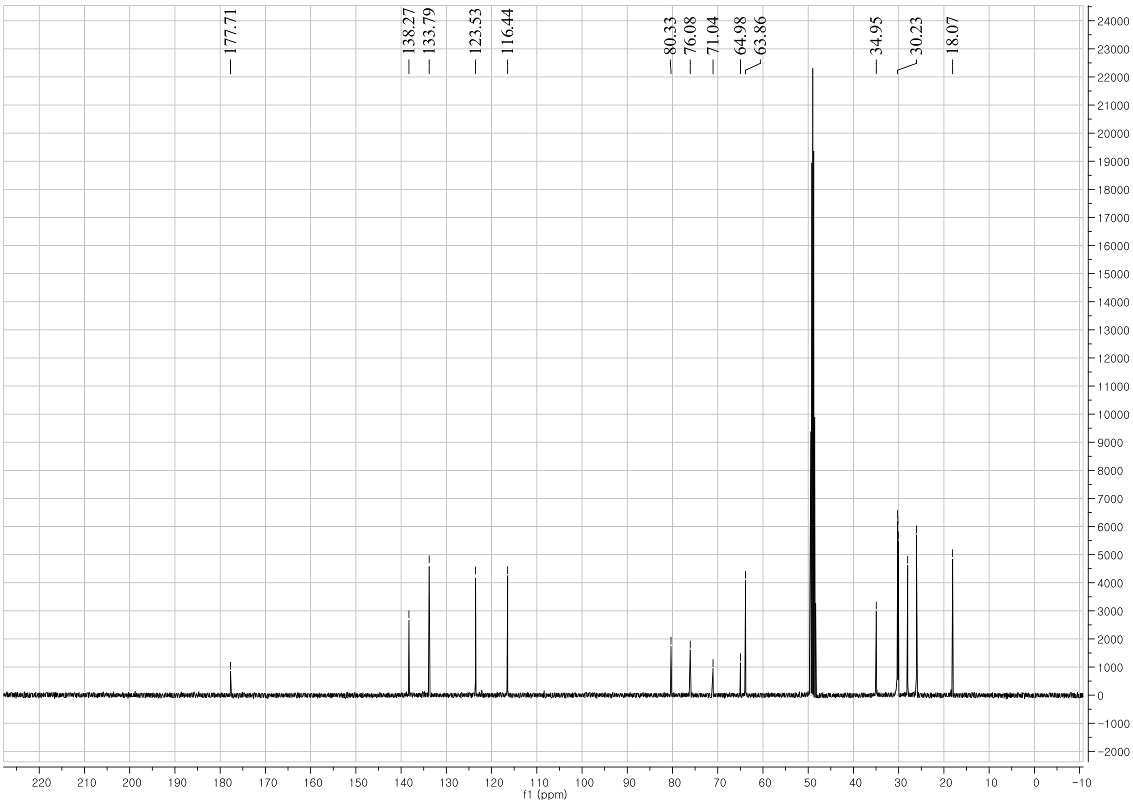


**Supplemental Figure 3.** $^{13}$C-NMR spectrum of (9*Z*, 16*S*)-16-Hydroxy-9,17-octadecadiene-12,14-diynoic acid (100 MHz, $CD_3OD$).

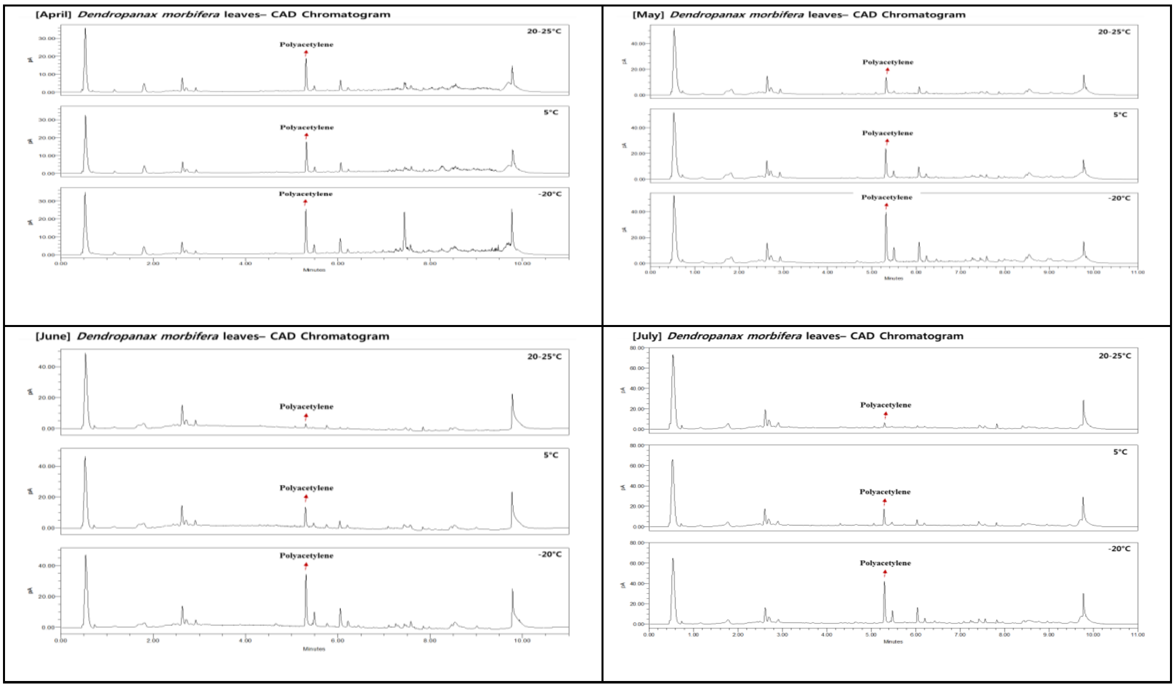


**Supplemental Figure 4.** UPLC-CAD chromatogram of the dry leaves in room temperture (20–25°C) on daylight without direct sunlight (upper), the leaves stored in a refrigerator at 5°C in darkness (middle), and the leaves stored in a refrigerator at -20°C in darkness (bottom).
